# Mechanical Fault Diagnosis of High Voltage Circuit Breakers Utilizing EWT-Improved Time Frequency Entropy and Optimal GRNN Classifier

**DOI:** 10.3390/e20060448

**Published:** 2018-06-07

**Authors:** Bing Li, Mingliang Liu, Zijian Guo, Yamin Ji

**Affiliations:** HLJ Province Key Lab of Senior-Education for Electronic Engineering, Heilongjiang University, Harbin 150080, China

**Keywords:** HV circuit breakers, mechanical fault diagnosis, empirical wavelet transform, improved time-frequency entropy, generalized regression neural network, loop traversal method

## Abstract

The mechanical fault diagnosis results of the high voltage circuit breakers (HVCBs) are mainly determined by the feature vector and classifier used. In order to obtain more remarkable characteristics of signals and a robust classifier which is suitable for small sample classification, in this paper, a new mechanical fault diagnosis method is proposed. Firstly, the vibration signals of HVCBs are collected by a designed acquisition system, and the noise of signals is eliminated by a soft threshold de-noising method. Secondly, the empirical wavelet transform (EWT) is adopted to decompose the signals into a series of physically meaningful modes, and then, the improved time-frequency entropy (ITFE) method is used to extract the characteristics of the vibration signals. Finally, a generalized regression neural network (GRNN) is used to identify four types of vibration signals of HVCBs, while the smoothing parameter δ of GRNN is optimized by a loop traversal method. The experimental results show that by using this optimal classifier for fault diagnosis, the proposed fault diagnosis method has the better generalization performance and the recognition rate of unknown samples is over 95%, and the signal features obtained by the ITFE method are more significant than those of the traditional TFE method.

## 1. Introduction

As an important switchgear in the power system, high voltage circuit breakers (HVCBs) have dual responsibilities of controlling and protecting the power grid. When HVCB failures occur and lead to abnormal outages, there will be economic losses and the people’s lives will be affected, therefore, it is urgent to study the fault diagnosis of HVCBs, so as to maintain the power grid over time. Studies show that many HVCB faults are caused by mechanical components [1,2]. Over the past decade, most HVCB fault diagnosis studies were about the mechanical vibration signals [2,3,4,5,6,7,8,9,10,11,12,13,14] and all these studies could be successfully applied to a certain extent because the vibration signals produced by mechanical components often provide abundant dynamic information about mechanical system condition.

The vibration signal of HVCBs is a transient non-stationary nonlinear time series, with a maximum frequency usually thought to be about 10 kHz [4]. The vibration events with frequencies greater than 10 kHz are very few, so these components can be ignored. The traditional physical instruments to collect these vibration signals are often characterized by single function and high cost. In this study, different from traditional physical instruments, a vibration signal acquisition system for HVCBs based on a “virtual instrument” is designed. This method is more flexible for signal acquisition. Through graphical (LabVIEW) programming (LabVIEW 2014, National Instruments, Austin, TX, USA), the “virtual instrument” can work like a real physical instrument, and many studies [15,16,17] now use LabVIEW for signal acquisition.

Transient non-stationary nonlinear time series are usually processed and analyzed by time-frequency methods. Since Hung et al. [18] proposed the empirical mode decomposition (EMD) in 1998, adaptive mode decomposition has attracted many mechanical fault diagnosis scholars’ attention [7,19], with variants such as local mean decomposition (LMD) [20], ensemble empirical mode decomposition (EEMD) [21,22], iterative filtering (IF) [23], empirical wavelet transform (EWT) [24], variational mode decomposition (VMD) [25], nonlinear mode decomposition (NMD) [26], adaptive local iterative filtering (ALIF) [27], etc. Although EMD and EEMD do not need any a priori basis to match the signal characteristic, they lack rigorous a mathematical formulation and always generate extra intrinsic modal function (IMF) components. Some studies therefore select the main IMFs for signal analysis [7,28,29,30], but an effective criterion for selecting IMFs is still lacking. Huang et al. [9,10] used LMD and VMD to decompose the vibration signals of HVCBs, respectively. However, in practical applications, LMD sometimes also suffers the same mode mixing shortcomings as EMD does [10], while for the VMD method, how to determine the number of IMFs is a difficult issue. NMD and ALIF are two new signal decomposition methods, which have not been widely applied in machinery fault diagnosis until now. The NMD method suffers from a high computational burden, and the NMD simulations take a lot of time, besides, because of the nature of NMD algorithm, many signals couldn’t extract a mode. As for the ALIF method, some signals that contain a lot of noise cannot be decomposed effectively. In view of the shortcomings of the above signal decomposition methods, the EWT method is adopted in this paper.

EWT is a new signal adaptive decomposition method, widely used in the field of mechanical fault diagnosis and its performance has been widely recognized [2,31,32]. Because it has the theoretical framework of wavelet transforms, and it can decompose the signals into a series of physically meaningful modes, the mode aliasing problem of EMD method can be well solved.

In the process of extracting the mechanical fault features of HVCBs, there are two commonly used feature extraction methods. The first method is the envelope energy entropy method [3,7], where the envelopes of IMF components are divided by means of equal time or equal energy, and then energy entropies are calculated as the characteristics. The second method is the time frequency entropy method (TFE) [2,4,11]. However, for the first method, the time-frequency distribution information of the original signal energy is lost to a certain extent, and it is easily affected by false modalities [2]. For the TFE method, at present, wavelet TFE (WTFE) has been widely used for signal feature extraction of HVCBs [4,11], however, the wavelet transform has frequency aliasing problems [33] and the wave base is hard to choose. Besides, in the time-frequency plane division process of the TFE method, the time-frequency plane is often divided in an even way [2,11], and the characteristics of the signal therefore cannot be well extracted. In view of the above shortcomings, in this paper, combined with EWT, The EWT-ITFE method therefore is proposed.

Finally, a suitable classifier is needed for fault diagnosis. The artificial neural network (ANN) [3,6,34] and support vector machine (SVM) methods [2,10,11] are the most commonly used in fault diagnosis of HVCBs. Thus, Sun et al. [3] used a back propagation neural network (BPNN) to diagnose HVCB faults, Yang et al. [34] used a probabilistic neural network (PNN) [35] to diagnose the HVCB faults and Huang et al. [10,11] used SVM to diagnose HVCB faults. However mechanical vibration samples of HVCBs are difficult to obtain, and BPNN is not suitable for the classification of small data samples. Although PNN and SVM are suitable for small sample datasets, the smoothing parameter δ of PNN has a great influence on the recognition results, and the penalty coefficient *C* and kernel function parameter *g* of SVM are also the same, while in the field of HVCB mechanical fault diagnosis, few people have studied the optimization of classifier parameters.

The generalized regression neural network (GRNN) is a supervised learning network, developed from the concept of the radial basis function neural network (RBFNN) [36]. Compared with BPNN and RBFNN, it has the advantages of fast training speed, strong approximation ability and high simulation accuracy [37]. GRNN is suitable for classification and prediction of small sample dataset. Li et al. [38] applied GRNN to fault diagnosis of rolling bearings, in which good results were achieved. However, like PNN, GRNN also has smoothing parameter δ that needs to be adjusted, where the smoothing parameter δ is the parameter contained in the transfer function of GRNN, and its value also called “spread”. In the process of mechanical fault diagnosis of HVCBs, people often ignore the influence of classifier parameters on the diagnosis results, thus, combined with K fold cross validation (K-CV), the loop traversal method is used to optimize the smoothing parameters δ. The loop traversal method can traverse all possible parameter values, then choose the best “spread” based on the principle of minimum mean square error (MSE).

The main contribution of this paper is to propose a novel mechanical fault diagnosis method for HVCBs based on EWT, ITFE and the optimal GRNN, where the optimal GRNN classifier is established by using the loop traversal method and K-CV. Firstly, a signal acquisition system is designed in LabVIEW, and the soft threshold de-noising is utilized to de-noise the signals. Then, after extracting the physically meaningful modes by the EWT method, and the signal features are calculated by the ITFE method. Finally, the optimal GRNN classifier is employed to identify the unknown samples.

## 2. Signal Acquisition and Fault Diagnosis Process

### 2.1. Experimental Materials

The data acquisition cards of the National Instruments (NI) Company are widely used for the mechanical vibration signal acquisition. They have the following advantages: flexible in use, high sampling rate, and a large number of ports. In this paper, the type of HVCB is ZW-12G/630-20 (Yueqing Wei Chuan Electric Co., Ltd., Zhejiang, China), so before we collect its vibration signals, we have to consider the frequency range of the vibration signals and the sampling rate of devices. The NI USB6002 is a data acquisition card with a maximum sampling rate of 50 kS/s, and an ADC resolution of 16 bits, which can extend external trigger circuits. These advantages are suitable for instantaneous signal acquisition in this experiment. Besides the NI USB6002 and trigger circuit, a LC0159 piezoelectric acceleration sensor (LANCE TECHNOLOGIES INC., Hebei, China), DC stabilized voltage source and an AFT-0931 signal conditioner (Electronic Technology Co., Ltd., Shijiazhuang, Hebei, China) are applied to build the vibration signal acquisition system for the HVCB. The frequency range of the LC0159 piezoelectric acceleration sensor is 1–12,000 Hz, and the range and sensitivity are 500 g, 10 mV/g respectively, while the frequency response and gain of the AFT-0931 signal conditioner are 0.5–45 kHz, and 5, respectively.

### 2.2. Design of Acquisition System

**Figure 1:**
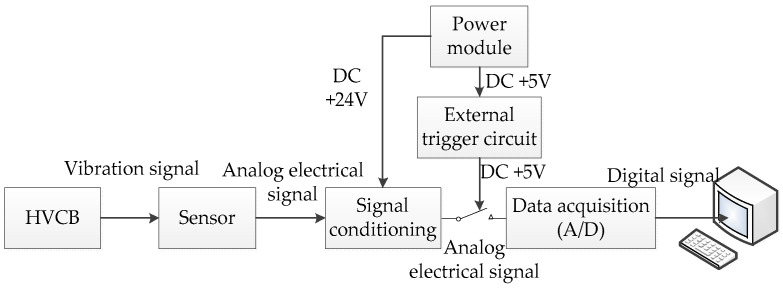
Design framework of the entire signal acquisition system. HVCB: high voltage circuit breaker; DC: direct-current.

In order to collect the transient non-stationary nonlinear vibration signals of HVCBs in real-time, in this paper, firstly, the LC0159sensor is used to transform the vibration signals into analog electrical signals. Secondly, the analog electrical signals are input into the AFT-0931 signal conditioner, and the filtering and amplification process of the analog electrical signals is then completed in the signal conditioner. In addition, the signal conditioner can prevent shock signals with big amplitude from damaging the host computer. Finally, the analog electrical signal is input into the NI USB6002 for AD conversion, and the AD conversion process will be assisted by the trigger circuit. The design framework of the entire signal acquisition system is shown in Figure 1. The trigger circuit triggers the NI USB6002 by +5 V high level, and when the HVCB vibration signals occur, the NI USB6002 will be triggered to work, otherwise, the host computer acquisition system will wait for the actions of the HVCB, and the NI USB6002 therefore will not work. This is very suitable for the acquisition of instantaneous signals.

### 2.3. Fault Diagnosis Process

**Figure 2:**
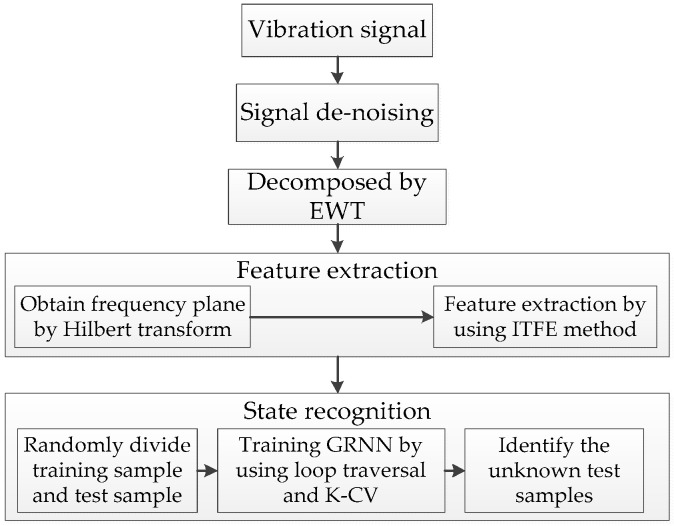
Flow chart of fault diagnosis. EWT: empirical wavelet transform; ITFE: improved time frequency entropy; GRNN: generalized regression neural network; K-CV: K fold Cross validation.

The new fault diagnosis method proposed in this paper mainly consists of three steps: signal decomposition, feature extraction and state recognition. In the signal decomposition step, the EWT method is adopted. In the feature extraction step, firstly, according to the modes decomposed by EWT, we use the Hilbert transform to calculate the time frequency plane of the signals. Secondly, according to the frequency domain features and time domain features of the signals, we segment the time frequency plane, then calculate the signal features by the ITFE method. In the state recognition step, firstly, all samples are randomly divided into a training set and test set, then the loop traversal and K-CV methods are used to find the best “spread” of the GRNN classifier. Finally, we use this optimal classifier to classify the test samples. The whole fault diagnosis process in this paper is shown in Figure 2.

## 3. EWT

### 3.1. EWT Decomposition Theory

**Figure 3:**
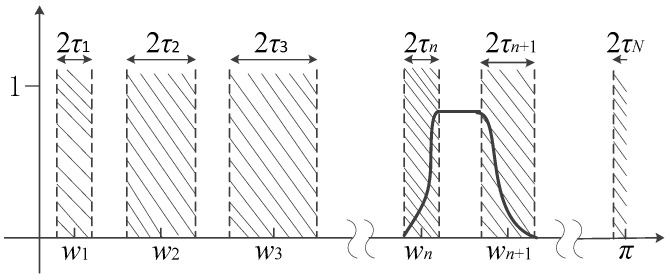
Division of the Fourier spectrum.

Nonlinear nonstationary signals are usually characterized by time-varying amplitudes and frequencies. To better analyze these signals, Gilles recently proposed the EWT method [24]. The main idea of EWT is to extract the different amplitude-modulated and frequency-modulated (AM-FM) components of a signal by designing a series of appropriate wavelet filter banks. It’s considered that the AM-FM components of a signal have a tightly supported Fourier spectrum, therefore, extracting the different modes is equivalent to segmenting the frequency axis of the Fourier spectrum and applying some filtering corresponding to each detected support. The detailed process of the EWT decomposition is as follows: firstly, we normalize the frequency range of the Fourier spectrum of the signal to the interval [0, π] (the unit is rad/s) and then adaptively segment the frequency axis into *N* contiguous subintervals, thus *N* + 1 boundaries are needed, where boundaries $\omega_{0}$ = 0 and $\omega_{N}\;=\;\pi$ are two boundaries, therefore we need to find *N* − 1 extra boundaries. Let $\omega_{n}$ to be the boundary of the adjacent interval, the choice of $\omega_{n}$ is the midpoint of two adjacent maxima of the Fourier spectrum, then each subinterval can be denoted as $\Lambda_{\lambda }\;=$ [$\omega_{\lambda -1}{,\omega }_{\lambda }$], where λ = 1, 2, …, *N*, thus $U_{\lambda =1}^{N}\Lambda_{\lambda }$ = [0, π], the segmentation of the Fourier axis is shown in Figure 3, the gray hatched areas are defined as transition phase $T_{n}$, its width is ${2\tau }_{n}$ and the center is $\omega_{n}$, and $\tau_{n}={\gamma \omega }_{n}$, where γ is coefficient, and 0 < γ < 1.

After determining the division interval $\Lambda_{\lambda }$, the empirical wavelets are defined as bandpass filters on each $\Lambda_{\lambda }$. In order to realize this idea, according to the construction methods of Littlewood-Paley and Meyer’s wavelet [39], similarly, the empirical scaling function ${\hat{\phi }}_{n}(\omega )$ is defined as follows:

(1) $${\hat{\phi }}_{n}(\omega )=\{\begin{array}{c} \begin{array}{c} 1,\;\left|\omega \right|\leq \omega_{n}-\tau_{n}\\ \cos\left(\frac{\pi }{2}\beta \left(\frac{1}{{2\tau }_{n}}\left(\left|\omega \right|-\omega_{n}+\tau_{n}\right)\right)\right),\\ \omega_{n}-\tau_{n}\leq \left|\omega \right|\leq \omega_{n}+\tau_{n} \end{array}\\ 0,\;\mathrm{other} \end{array}$$

while the empirical wavelets function ${\hat{\psi }}_{n}(\omega )$ are defined as:

(2) $${\hat{\psi }}_{n}(\omega )=\{\begin{array}{c} 1,\;\omega_{n}{+\tau }_{n}\leq \left|\omega \right|\leq \omega_{n+1}-\tau_{n+1}\\ \cos(\frac{\pi }{2}\beta \left(\frac{1}{{2\tau }_{n+1}}\left(\left|\omega \right|-\omega_{n+1}+\tau_{n+1}\right)\right)),\;\\ {\;\omega }_{n+1}-\tau_{n+1}\leq \left|\omega \right|\leq \omega_{n+1}+\tau_{n+1}\\ \sin\left(\frac{\pi }{2}\beta \left(\frac{1}{{2\tau }_{n}}\left(\left|\omega \right|-\omega_{n}+\tau_{n}\right)\right)\right)\\ \omega_{n}-\tau_{n}\leq \left|\omega_{n}\right|\leq \omega_{n}+\tau_{n}\\ 0,\;\mathrm{other} \end{array},$$

where $\tau_{n}={\gamma \omega }_{n}$, and in order to meet tight frame conditions, let γ < $\min_{n}\left(\left(\omega_{n+1}-\omega_{n}\right)/\left(\omega_{n+1}+\omega_{n}\right)\right)$. Function β(x) is an arbitrary $C^{k}$ function, and for ∀x∈[0,1]:

(3) β(x)+β(1−x)=1,

and usually:

(4) $$\beta \left(x\right)=x^{4}\left(35-84x+{70x}^{2}-{20x}^{3}\right)\;,$$

According to the definition of ${\hat{\phi }}_{n}(\omega )$ and ${\hat{\psi }}_{n}(\omega )$, similar to the construction method of classic wavelet, we can now define the empirical wavelet transform. The detail coefficient is defined by the inner product of the signal f(t) and the empirical wavelet function $\psi_{n}\left(t\right)$:

(5) $$\omega_{f}^{\varepsilon }(n,t)=\langle f\left(t\right),\psi_{n}(t)\rangle =\int f(\tau )\bar{\psi_{n}(\tau -t)}d\tau =F^{-1}(f\left(\omega \right){\hat{\psi }}_{n}\left(\omega \right))\;,\;$$

while the approximate coefficient is defined by the inner product of the signal f(t) and the scaling function $\phi_{1}\left(t\right)$:

(6) $$\omega_{f}^{\varepsilon }\left(0,t\right)=\langle f(t),\phi_{1}(t)\rangle =\int f(\tau )\bar{\phi_{1}(\tau -t)}\;d\tau =F^{-1}(f\left(\omega \right){\hat{\phi }}_{1}\left(\omega \right))\;,\;$$

where ${\hat{\psi }}_{n}\left(\omega \right)$ and ${\hat{\phi }}_{1}\left(\omega \right)$ are the function defined by Equations (1) and (2) respectively, and $F(\psi_{n}\left(t\right))={\hat{\psi }}_{n}\left(\omega \right)$, $F(\phi_{1}\left(t\right))={\hat{\phi }}_{1}\left(\omega \right)$, symbol F denotes the Fourier Transform, $\bar{\psi_{n}(\tau -t)}$ and $\bar{\phi_{1}(\tau -t)}$ are the complex conjugate of $\psi_{n}(\tau -t)$ and $\phi_{1}(\tau -t)$ respectively. Finally, reconstructing signal f(t) by detail coefficient and approximate coefficient:

(7) $$f(t)=\omega_{f}^{\varepsilon }*\phi_{1}(t)+\sum_{n=1}^{N}\omega_{f}^{\varepsilon }(n,t)*\psi_{n}(t)=F^{-1}({\hat{\omega }}_{f}^{\varepsilon }(0,\omega ){\hat{\phi }}_{1}(\omega )+\sum_{n=1}^{N}{\hat{\omega }}_{f}^{\varepsilon }(n,\omega ){\hat{\psi }}_{n}(\omega ))$$

where symbol * denotes convolution operation, and $F(\omega_{f}^{\varepsilon }(0,t))={\hat{\omega }}_{f}^{\varepsilon }(0,\omega )$, $F(\omega_{f}^{\varepsilon }(n,t))={\hat{\omega }}_{f}^{\varepsilon }(n,\omega )$, following this mathematical form, the empirical mode $f_{k}$ (*k* = 0,1,2…*N* − 1) is defined as:

(8) $$f_{k}\left(t\right)=\{\begin{array}{c} \omega_{f}^{\varepsilon }\left(0,t\right)*\phi_{1}\left(t\right),\;k=0\\ \omega_{f}^{\varepsilon }\left(k,t\right)*\psi_{k}(t),k=1,2\ldots N-1 \end{array}\;,\;$$

### 3.2. Determining the Segment Number N of EWT

The segment number *N* (or number of mode) should be predefined in the EWT method, as the appropriate value of *N* can reflect the physically meaningful components of signals. Gilles [29] gives a threshold method to determine the value of *N*, the basic idea being that the most important maxima in the amplitude of the Fourier transform of the input signal (corresponding to the center of each desired Fourier segments) are significantly larger than the other existing maxima. Let ${\left\{M_{i}\right\}}_{k=1}^{K}$ be the amplitude of the detected maxima in the frequency range. Then arrange $M_{i}$ in descending order ($M_{1}\geq M_{2}\geq M_{3}\ldots \geq M_{K}$) and normalized it in interval [0, π] (rad/s). In practice, we can keep all maxima which are larger than a predefined threshold $M_{K}+\alpha \left(M_{1}-M_{K}\right)$, where the value of α is around 0.3 and 0.4. Now there are two cases:

(1) K≥N, in this case, the algorithm finds enough maxima in Fourier spectrum, then just keep the first *N* − 1 maxima.
(2) K<N, in this case, the signal has less modes than expected, then keep all the detected maxima and reset *N* to the appropriate value.

### 3.3. Simulation Signal Decomposition by EWT Method

#### 3.3.1. Modeling of Classical Simulation Signal

In the closing process of HVCBs, an impact or friction of the mechanical components in the operating mechanism will generate a vibration event, then, each sub-vibration event superimposes on the measuring point of the sensor, and the final measured signal is therefore formed. In the field of HVCB fault diagnosis, many researchers adopt the following signal as simulation signal for HVCBs [2,4,10,40]:

(9) $$f\left(t\right)=\overset{n}{\underset{i=1}{\sum }}A_{i}e^{-\alpha_{i}\left({t-t}_{i}\right)}{\sin(2\pi f}_{i}\left({t-t}_{i}\right))u\left({t-t}_{i}\right)+\omega \left(t\right),$$

where *n* is the number of vibration events, $A_{i}$ is maximum amplitude of the *i*-th vibration event, $\alpha_{i}$ is the attenuation coefficient of the amplitude, *u*(*t*) is the unit step signal, $t_{i}$ is the starting time of each vibration component, $f_{i}$ is the main frequency of each vibration component. ω(t) is white noise and $\omega (t)\sim N\left(0,0.01^{2}\right)$. All parameters of the simulation signal are set as shown in Table 1.

**Figure 4:**
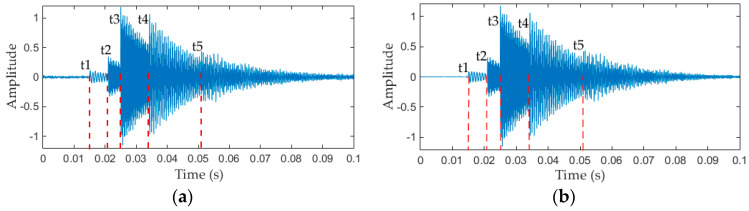
(**a**) Time domain waveform of simulated signal; (**b**) De-noised signal obtained by soft threshold method.

Matlab (Matlab R2014b, MathWorks, Natick, MA, USA) is used to generate the simulation signal f(t), and the sampling frequency is set to 40 kS/s, sampling time is set to 0.1 s, 4000 sampling points are collected in total. The time domain waveform of the original simulation signal f(t) and the de-noised signal are shown in Figure 4a,b respectively. The simulation signal denoted in Figure 4a is simple, but is representative because of its multi-component nature and non-stationarity.

#### 3.3.2. Simulation Signal Decomposition

**Figure 5:**
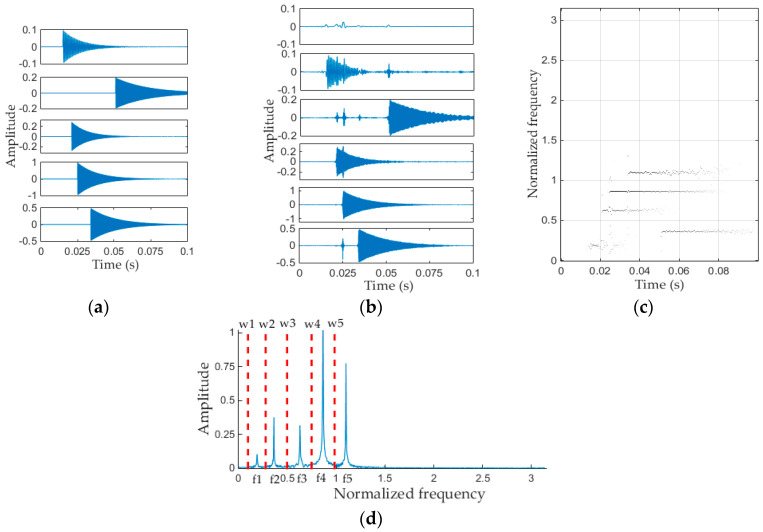
(**a**) Vibration components of simulation signal; (**b**) IMFs decomposed by EWT method; (**c**) Time-frequency plane obtained by EWT method; (**d**) Partitioning of the Fourier axis. IMFs: Intrinsic modal functions.

**Figure 6:**
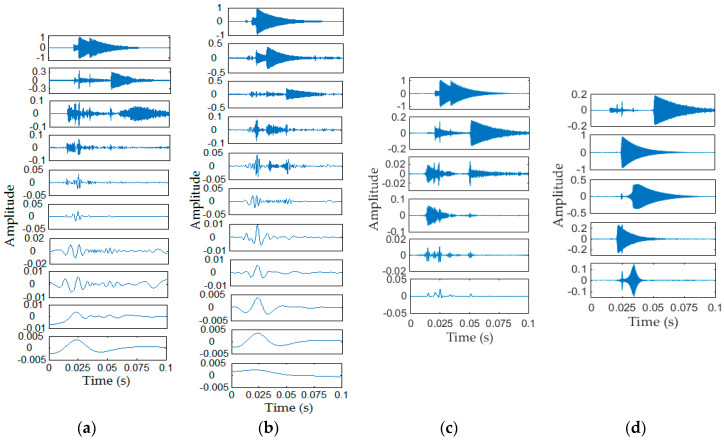
(**a**) IMFs decomposed by EMD; (**b**) IMFs decomposed by EEMD; (**c**) IMFs decomposed by IF; (**d**) IMFs decomposed by VMD. EMD: empirical mode decomposition; EEMD: ensemble empirical mode decomposition; VMD: variational mode decomposition.

The EMD and EEMD methods have been widely used in mechanical fault diagnosis in past decades [7,19,22,28]. In recent years, the emerging methods mainly include IF [23], VMD [25], NMD [26] and ALIF [27], etc. The pros and cons of each method have been discussed in detail in the Introduction section. Here, we mainly compare the performances of EMD, EEMD, IF and VMD methods to decompose the simulation signal (the signal in Figure 4). The performances of these five method are shown in Figure 5 and Figure 6, where the value of *N* in EWT method is 6, the value of *K* in VMD method is 5, and the number of IMFs of IF method is set to 5, the noise standard deviation, number of realizations, maximum number of sifting iterations of EEMD method are set to 0.01, 100, 20,000, respectively, and the EMD method uses default parameters.

It can be see that the Fourier axis is adaptively divided into six segments in Figure 5d, and correspondingly, the simulation signal is decomposed into six modes by the EWT method, as shown in Figure 5b. Each mode (in Figure 5b, from top to bottom) is the filtering result of applying a band-pass filter on the interval [0, $\omega_{1}$], [$\omega_{1}$, $\omega_{2}$], [$\omega_{2}$, $\omega_{3}$], [$\omega_{3}$, $\omega_{4}$], [$\omega_{4}$, $\omega_{5}$], [$\omega_{5}$, π], respectively. We can see from Figure 5b that the second to sixth modes are mostly the same as the corresponding vibration component in Figure 5a. However the first IMF component decomposed by the EWT method corresponds to the first segment (interval [0, $\omega_{1}$]) in Figure 5d, and it mainly caused by noise, rather than any real physically meaningful components. Because the amplitude of the first mode is very small, therefore, it can be ignored. That is, the EWT method can decompose simulated vibration signals thoroughly, and each physically meaningful components of the simulated signal can be obtained by using the EWT method. Figure 5c is the time-frequency plane, and the frequency components of simulation signal in the graph are clearly visible.

Conversely, in Figure 6a,b, we can see that more than 10 modes are obtained by EMD and EEMD method. The modes decomposed by EMD method have serious modal aliasing problem, especially for the first mode. Although EEMD can eliminate modal aliasing to a certain extent, but compare with EWT method, each mode obtained by EEMD is still unclearly. IF is better than EMD in some aspect, such as it overcomes the shortcoming of sensitivity to the singularities, however it shows almost the same performance as EMD with modal aliasing in this study, just as shown in Figure 6c, 6 modes are obtained, where the last one is the trend and the remaining modes are actual IMFs, however the first mode contains several vibration components. In Figure 6d, due to some parameter settings, the fifth mode obtained by VMD is not physically meaningful mode, while the remaining four modes are relatively close to original vibration components. The VMD method is generally better than other methods (like EMD, EEMD and IF) as shown in Figure 6.

In summary, the characteristics (such as starting time and spectrum) of each mode obtained by EMD, EEMD, IF and VMD are unclearly or untrue to a certain extent, i.e., they are not the best way for feature extraction of nonstationary nonlinear signals. Therefore, the EWT method is used in this paper.

## 4. Feature Extraction by Improved Time-Frequency Entropy Method

### 4.1. Calculation of Time-Frequency Plane

The instantaneous frequency of the original multi-component signal at a certain moment is not physically meaningful, because signal’s multi-component nature. By introducing the Hilbert transform, the corresponding analytic signals can be constructed from the original signal, and then the characteristics of original signal can be obtained, such as instantaneous amplitude, instantaneous frequency and time-frequency spectrum. In order to make instantaneous frequency meaningful, the signal for Hilbert transform should be single component signal. While the modes decomposed by EWT method are single component, combining EWT and Hilbert transform, the time-frequency plane with meaningful instantaneous frequency can be obtained.

For a single component signal x(t), the Hilbert transform is defined as follows:

(10) $$\hat{x}(t)=\frac{1}{\pi t}*x(t)=\frac{1}{\pi }\int_{-\infty }^{+\infty }\frac{x(t)}{t-\tau }dt,$$

where * denotes convolution operation. Then the analytic signal g(t) is defined as:

(11) $$g(t)=x(t)+j\hat{x}(t)=A(t)e^{j\varphi (t)}=A(t)e^{j\int_{0}^{T}\omega (t)dt}$$

where $A(t)=\sqrt{{x(t)}^{2}+{\hat{x}(t)}^{2}}$ is instantaneous amplitude of g(t), $\varphi (t)=arctan\frac{\hat{x}(t)}{x(t)}$ is phase of g(t), and $\omega (t)=\frac{d\varphi \left(t\right)}{dt}$ is the instantaneous frequency of single component signal x(t).

According to Equations (7) and (8), when Hilbert transform is performed on each IMF component $f_{k}\left(t\right)$, the original signal f(t) can be denoted as:

(12) F(t)=∑k=0N−1fk(t)=Re∑k=0N−1gk(t)=Re∑k=0N−1Ak(t)ejφk(t)=Re∑k=0N−1Ak(t)ej∫0Tωk(t)dt , 

That is to say, the original signal f(t) can be expressed as the bivariate functions of time *t* and instantaneous frequency ω, i.e., f(t)=H(ω,t), where H(ω,t) is the time-frequency plane and the integral of H(ω,t) to time *t* is marginal spectrum h(ω):

(13) $$h\left(\omega \right)=\int_{0}^{T}H\left(\omega ,t\right)dt,$$

while the integral of $H^{2}\left(\omega ,t\right)$ to frequency ω is instantaneous energy density IE(t):

(14) $$IE\left(t\right)=\int_{\omega }H^{2}\left(\omega ,t\right)d\omega$$

### 4.2. Improved Time-Frequency Entropy Method

**Figure 7:**
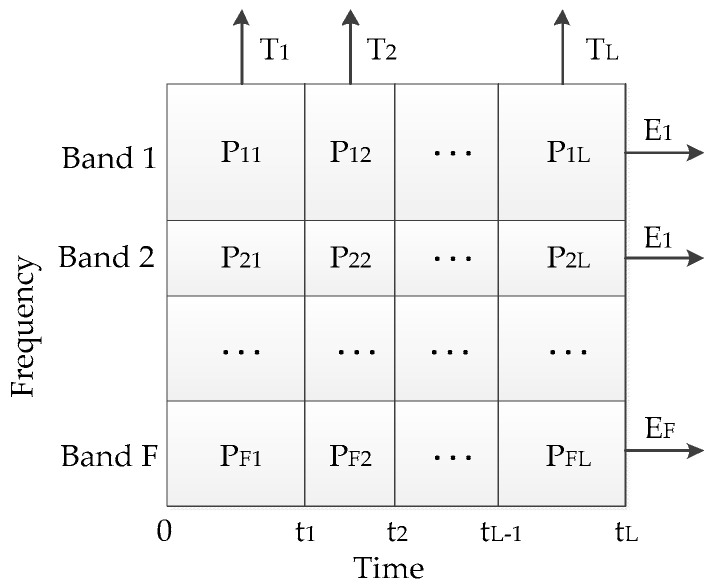
Division of the time-frequency plane.

Wavelet time-frequency entropy (WTFE) method is one of feature extraction method of vibration signal of HCVBs [2,4]. In order to calculate the time frequency entropy, the whole time-frequency plane needs to be divided into several sub-planes. However, it is not enough to reflect the characteristics of signal accurately by just dividing time-frequency plane equably. Therefore, inspired by the method of equal energy segmentation of signal envelope [3], in this paper, a new time-frequency plane division method is proposed, the main idea is as follows: for a standard normal state signal, firstly, divide the time-frequency plane’s time axis into *L* segments by equal energy segmentation. Then find *F* main frequency bands of the standard signal through the marginal spectrum, according to these frequency bands, divide the time-frequency plane’s frequency axis into *F* segments. Finally, record the position of each division time (0, $t_{1}$, …, $t_{L}$) and frequency band (band 1, band 2, …, band *F*), and apply this segmentation method to all signals. The segmentation schematic diagram of the time frequency plane is shown in Figure 7:

Where each sub-plane may not be the same size due to the new division method, and the *F* main frequency bands are determined by minimum of the marginal spectrum, i.e., *F* − 1 minimums of marginal spectrum are selected as the partition boundary in this paper.

When mechanical faults of HVCBs occur, the energy distribution along the time-frequency plane’s time axis and frequency axis will change, therefore, in order to measure this change, the information entropy is used in this paper. Firstly, the amplitude contribution (or energy distribution) of each sub-plane ($P_{11},P_{12},\ldots ,P_{FL}$) should be normalized to the interval [0, 1], i.e., satisfy the condition $0\leq P_{ij}\leq 1\;\left(i=1,2,\ldots ,F;\;j=1,2,\ldots ,L\right)$. In order to calculate time-frequency entropy $T_{j}$ and $E_{i}$ separately, there are two cases need to be discussed in this paper.

1. Along the time axis
  (15) $$P_{ij}=A_{ij}/\overset{L}{\underset{k=1}{\sum }}A_{ik},\;i=1,2,\ldots ,F;\;j=1,2,\ldots ,L\;,$$
  (16) $$E_{i}=-\overset{L}{\underset{j=1}{\sum }}P_{ij}{\log P}_{ij}\;,\;i\;=\;1,2,\ldots ,F\;,$$
2. Along the frequency axis
  (17) $$P_{ij}=A_{ij}/\overset{F}{\underset{q=1}{\sum }}A_{qj},i=1,2,\ldots ,F;\;j=1,2,\ldots ,L\;,$$
  (18) $$T_{j}=-\overset{F}{\underset{i=1}{\sum }}P_{ij}{\log P}_{ij},\;j=1,2,\ldots ,L\;,$$
  where $A_{ij}$ is the integral (for discrete signals, it is the sum) of the amplitude envelope of the corresponding sub-plane. Then the feature vector can be denoted as:
  (19) $$F_{v}=\left[T_{1},T_{2}{,\ldots ,T}_{L}{,E}_{1},E_{2}{,\ldots ,E}_{F}\right],$$
  where the value of $E_{i}$ describes the characteristics of signal in the frequency domain, while the value of $T_{j}$ describes the characteristics of signal in the time domain, thus the time migration of each vibration events can be detected by $T_{j}$. Therefore the proposed ITFE method can measure the features of signals from both frequency domain and time domain.

### 4.3. Steps of Feature Extraction

In summary, an improved time-frequency entropy (ITFE) method is proposed to extract HVCB vibration features in this paper. The main steps are now summarized as follows:

(1) Decompose the vibration signals of HVCB into a series of physically meaningful modes by EWT method.
(2) According to these mode components, calculate the time frequency plane and marginal spectrum of vibration signal by Hilbert transform.
(3) Divide the time frequency plane into *F* × *L* segments by using proposed ITFE method.
(4) Calculate the time-frequency entropy $T_{j}$ and $E_{i}$, and then construct the feature vectors $F_{v}=\left[T_{1},T_{2}{,\ldots ,T}_{L}{,E}_{1},E_{2}{,\ldots ,E}_{F}\right]$.

## 5. Optimal GRNN Classifier Design

**Figure 8:**
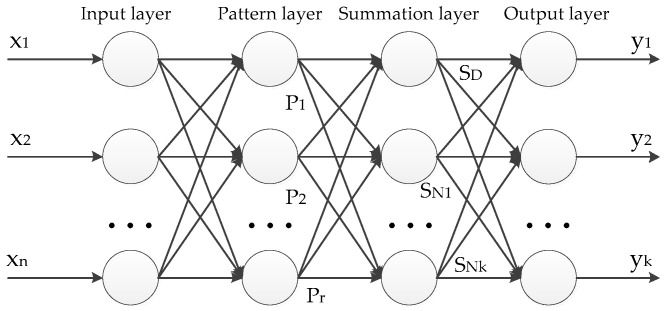
The structure diagram of generalized regression neural networks.

The generalized regression neural network (GRNN) was proposed by Donald F. Specht in 1991 [36]. It is suitable for solving small samples, nonlinear problems, and its structure is similar to radial basis neural network (RBFNN) [6], as shown in Figure 8:

It can be see that GRNN consists of four layers, i.e., input layer, pattern layer, summation layer and output layer. The number of input layer neuron is equal to the dimension *n* of input vectors ***X*** (assume that ***X*** = ${\left[x_{1},x_{2},\ldots ,x_{n}\right]}^{T}$), the input vectors pass directly to the pattern layer without any change. While the number of pattern layer neuron is equal to the total number *r* of training samples, and each pattern layer neuron corresponds to a training sample. The transfer function of pattern layer neuron *i* is

(20) $$P_{i}=\exp\left[-\frac{{\left(X-X_{i}\right)}^{T}\left(X-X_{i}\right)}{{2\sigma }^{2}}\right],i=1,2,3,\ldots ,r,$$

where $P_{i}$ is the output of pattern layer neuron *i*, X is the input vector of a text sample, $X_{i}$ is the corresponding training sample of neuron *i*, σ is the smoothing parameter that needs to be optimized, and the value of σ is always called “spread”. Equation (20) shows that the output of neuron *i* in pattern layer is related to the Euclidean distance between X and $X_{i}$. In the summation layer of GRNN, two summation methods exist:

(1) Summing all outputs of mode layers directly, i.e., the connection weight between each pattern layer neuron and summation layer neuron is 1. The transfer function can be denoted as:
  (21) $$S_{D}=\overset{r}{\underset{i=1}{\sum }}P_{i},i=1,2,3,\ldots ,r,$$
(2) Summing all outputs of mode layers with weight $W_{ij}$, the transfer function can be denoted as:
  (22) $$S_{Nj}=\overset{r}{\underset{i=1}{\sum }}y_{ij}P_{i},j=1,2,3,\ldots ,k,$$
  where $y_{ij}$ is the weight between *i*-th neuron of pattern layer and *j*-th neuron of summation layer.

In the output layer of GRNN, the number of neurons is equal to the dimension *k* of output vectors of learning sample. The estimation results $\hat{Y}(X)=[y_{1},y_{2},y_{3},\ldots y_{k}]$ can be calculated as:

(23) $$y_{j}=\frac{S_{Nj}}{S_{D}},j=1,2,\ldots ,k,$$

**Figure 9:**
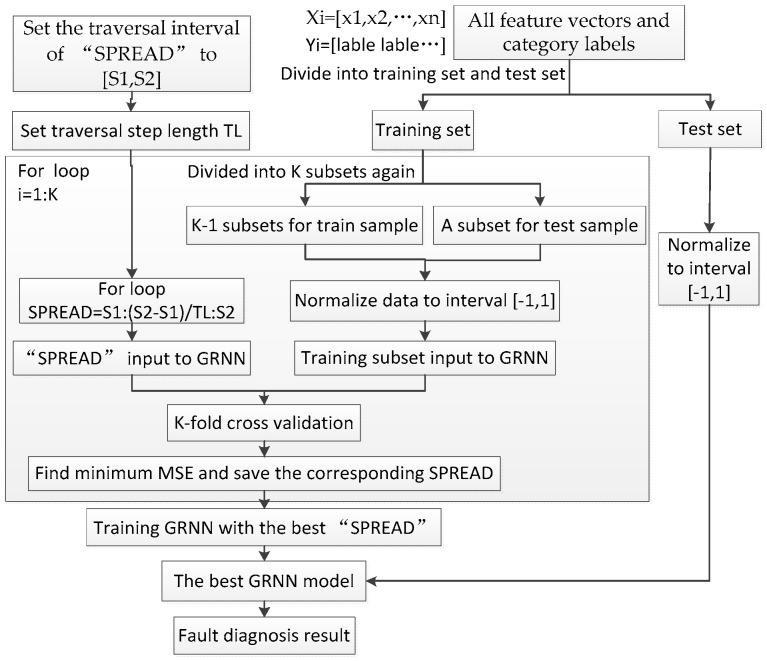
Whole design process of the classifier.

It can be seen from Equation (20) that the value of smoothing parameter σ (“spread”) in transfer function needs to be determined in advance. The larger “spread” is, the smoother the function approximation will be, in this case, GRNN may cause under-fitting results. When “spread”→0, the more accurate the function approximation will be, GRNN may cause over-fitting results, therefore, the network might not generalize well. In summary, the value of “spread” has a great influence on the performances of GRNN classifier, it is necessary to optimize the “spread”. In this paper, we use the loop traversal method to find the best parameter “spread”, then use K fold cross-validation (K-CV) to verify the rationality of the parameter “spread”, where the rationality of the parameters “spread” is measured by the MSE (MSE is a commonly used loss function in classification problems). The loop traversal method is similar to grid search (GS) method, the “spread” will traverse the entire value interval [$S_{1},S_{2}$] with the step length *TL*. Compared with the method of choosing “spread” by experience, the loop traversal method is more efficient. The whole design process of the classifier is shown in Figure 9.

## 6. Experimental Results and Analysis

### 6.1. Signal Collection

**Figure 10:**
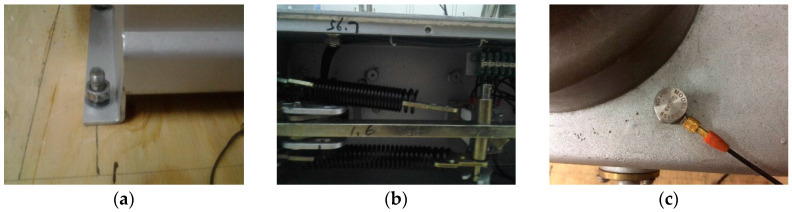
(**a**) Simulation of fault I; (**b**) Simulation of fault III; (**c**) Location of the sensor.

The vibration signals of HVCB are collected by designed acquisition system in Figure 1. In this paper, there are four mechanical operating states that have been simulated: Normal state (Normal), looseness of the base screw (Fault I), delay fault (Fault II), one of the energy storage spring shedding (Fault III). Among them, Fault I and Fault III are simulated just like Figure 10a,b respectively. While Fault II is mainly caused by delay action of the electromagnetic mechanism, and compare with the normal state, it only has time delay, therefore, here we use the LabVIEW program to achieve this type of fault, i.e., in normal condition, set a delay time to the acquisition system. In Figure 10c, it is the measurement position of sensor, the sensor is placed close to the operating mechanism.

**Figure 11:**
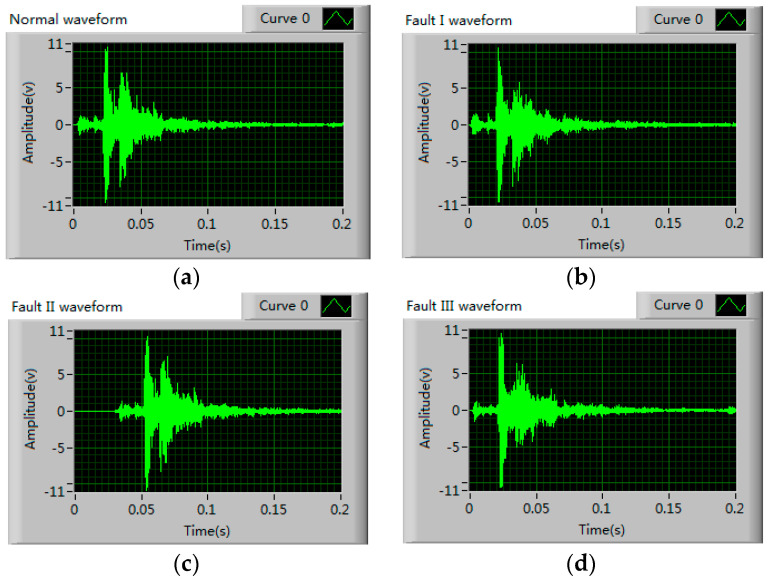
Time domain waveform of each mechanical state (**a**) Normal state; (**b**) Fault I; (**c**) Fault II; (**d**) Fault III.

In this paper, 30 samples per each mechanical state are collected by acquisition system, so there are 120 samples in total. Figure 11 shows the time domain waveform of Normal signal and three types of fault signals, the sampling frequency is 40 Ks/s, and the sampling time is 0.2 s. It can be seen in Figure 11 that the time domain waveform of normal state and fault II is very similar, the main difference between them is only a time delay. While relatively speaking, the other two kinds of faults are more different from normal conditions. These differences are mainly caused by the impact or friction of the each mechanical component of operating mechanism, when the mechanical fault occurs, the impact or friction process will change to a certain extent, therefore the instantaneous frequency, instantaneous amplitude and occurrence time of each vibration event will be different.

**Figure 12:**
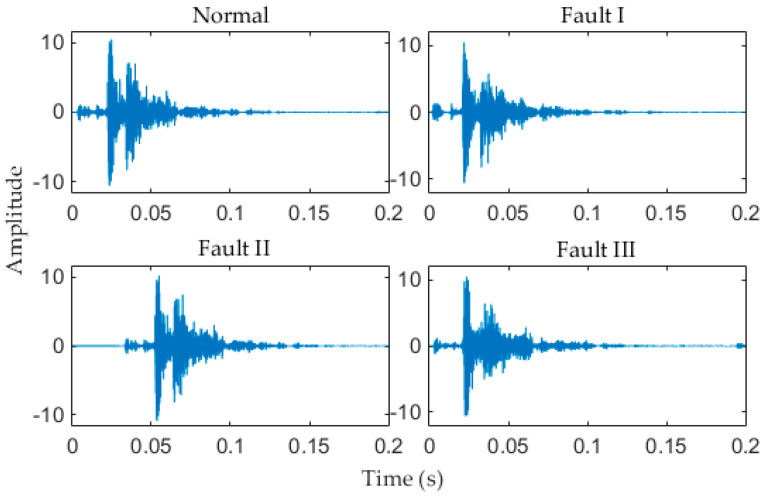
De-noised signal of four types of signals.

As mentioned previously, after the signal acquisition work is completed, signals need to be de-noised. Taking into account the smoothness of the de-noised signal, in this paper, the soft threshold de-noising method is applied in signal de-noising. The de-noised signal of four types of original signals are shown in Figure 12.

### 6.2. Signal Decomposition and Feature Extraction

**Figure 13:**
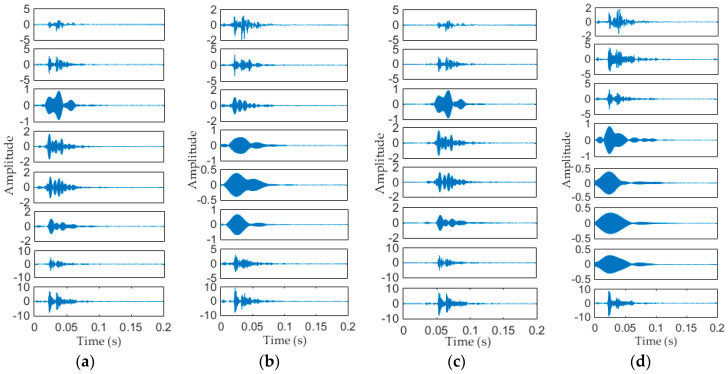
Vibration components of the four types of signals obtained by EWT method. (**a**) Normal state; (**b**) Fault I; (**c**) Fault II; (**d**) Fault III.

Different from simulation signal in Equation (9), the measured signals are more complicated. Here are 8 components that have been decomposed from measured signals, as shown in Figure 13, each mode component is a narrow band AM-AF signal. It can be seen that each mode component of Normal signal is similar to the Fault II, their main difference is a time delay. While the modes of Fault I, Fault III and Normal state are different from each other, i.e., the frequency distribution of the vibration events has changed, and this change is mainly caused by mechanical components.

**Figure 14:**
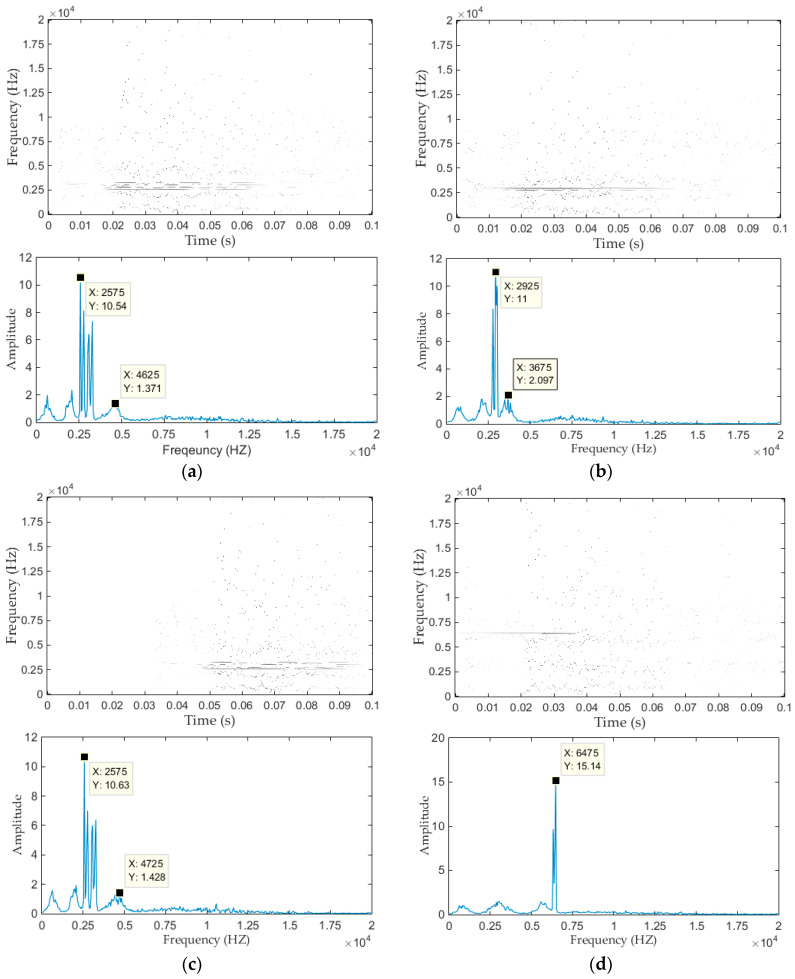
Time frequency plane and marginal spectrum of the four types of signals. (**a**) Normal state; (**b**) Fault I; (**c**) Fault II; (**d**) Fault III.

The time-frequency plane and marginal spectrum of the four types of vibration signals are shown in Figure 14. Where the time-frequency plane obtained in this paper is a matrix of 400 rows and 7889 columns, and the values of the elements in this matrices represent the instantaneous amplitude. It can be seen from Figure 14 that a lot of black spots exist in time-frequency plane, while the color intensity of these black spots measure the magnitude of instantaneous amplitude. Therefore, the time-frequency plane can reflect the instantaneous amplitude, instantaneous frequency and occurrence moment of the vibration signal, the characteristics of the signals therefore can be well extracted from the time-frequency plane.

Since the signals have a very small amplitude between 0.1 to 0.2 s, the time-frequency plane presents a large blank, for clarity, we only show the time frequency plane from 0 to 0.1 s (however, the whole time-frequency plane is still used to extract signal features). It can be seen from time-frequency plane that the frequency distribution of Normal, Fault I and Fault II states are similar, especially Normal and Fault II states, and the frequencies of them are concentrated around 3000 Hz, while the frequency distribution of Fault III are obviously different. Besides, there is a time delay between Fault II state and other three states. Therefore, it is necessary to divide the time-frequency plane according to the time-frequency distribution characteristics of the signals, rather than simply dividing the time-frequency plane evenly. All these characteristics can be further seen from the marginal spectrum.

According to Equation (13), the marginal spectrum can be obtained from the corresponding time frequency plane, it can be seen from marginal spectrum that the main frequency components of the measured signals (Normal, Fault I, Fault II) are concentrated in 0~4725 Hz, while for Fault III, Its maximum frequency component is around 6475 Hz. Besides, the marginal spectrum of the normal state signal is similar to Fault II, while other two types of faults are different from each other. Therefore, we need to distinguish between Normal and Fault II from other aspects, such as occurrence time of vibration events. In summary, in order to correctly identify the four types of vibration signals, we must pay attention to both time domain features and frequency domain features.

After the signal is decomposed by the EWT method, the ITFE method is adopted to extract the features of vibration signals. Firstly, select a standard normal state vibration signal from the collected signals. Then according to the minimum distribution of the marginal spectrum, and the number of mode components in Figure 13, horizontally divide the time-frequency plane into 9 segments, and according to the principle of equal energy segmentation, vertically divide the time-frequency plane into 10 segments, therefore, 9 × 10 sub-planes are obtained. Finally, record the division boundaries of this standard time-frequency plane and apply it to other signals. Here the choice of segment number is the result of multiple experiments, when segment number is too large, the features of signal are too sensitive to change, however, when the segment number is too small, the features of signal are not obvious enough.

The division boundaries of standard normal state vibration signal are recorded as shown in Table 2. In this paper, time-frequency plane is a matrix of 400 rows, 7998 columns, the “serial number” in Table 2 represents the row where the frequency boundary is located in the time-frequency plane. While for discrete time series, sampling points can be equivalent to time, i.e., *t* = 0 s corresponds to the first sampling point, while *t* = 0.2 s corresponds to the 8000th sampling point in this paper.

Compared with TFE method, the proposed ITFE method can divide the time-frequency plane more densely in the places where the signal energy or frequency bands are concentrated. In this paper, combined with EWT, the empirical wavelet transform-improved time frequency entropy (EWT-ITFE) feature extraction method is proposed. To validate the superiority of proposed EWT-ITFE method, EWT-ITFE, EWT-TFE and WTFE method are compared in this study. Where the WTFE method follows the feature extraction method in literature [11], and the db35 wavelet basis is chosen, while the decomposition levels of wavelet packet transform is 5, besides, the time-frequency plane is equally divided into 10 × 9 parts in this study, i.e., horizontally divide the time-frequency plane into 9 segments, and vertically divide the time-frequency plane into 10 segments (the same with EWT-TFE method).

**Figure 15:**
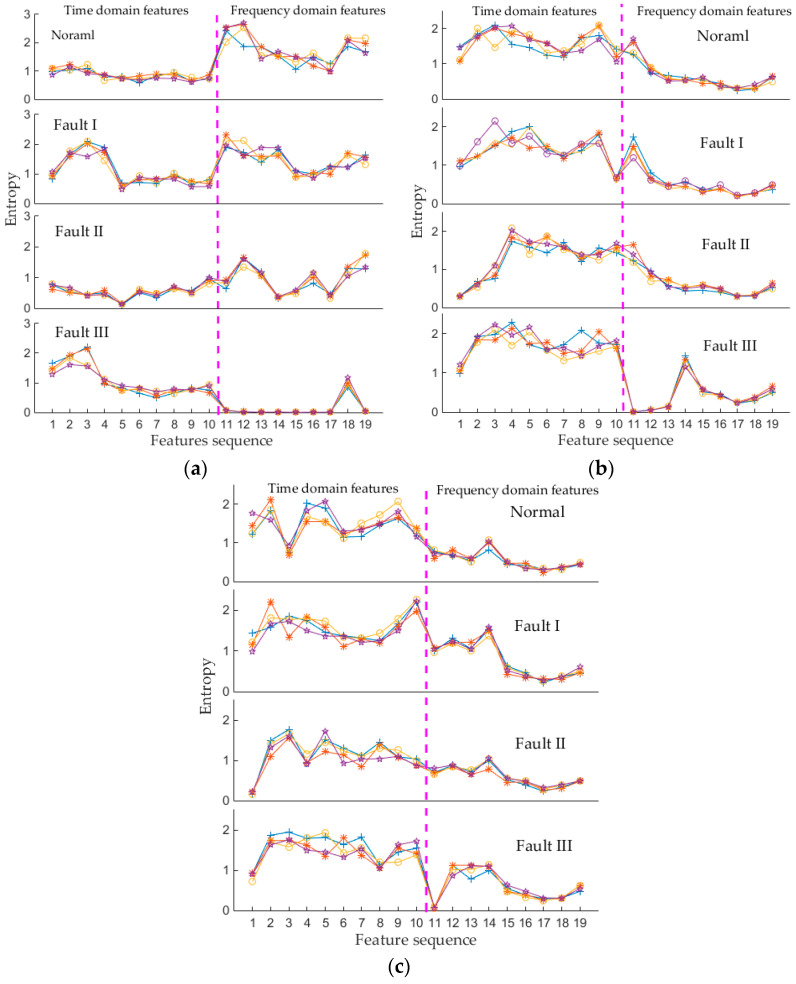
Feature distribution of four types of signals. (**a**) EWT-ITFE method; (**b**) EWT-TFE method; (**c**) WTFE method. WTFE: wavelet Time frequency entropy.

In order to describe the overall trend of the feature vectors of each type of signal, Figure 15 shows the EWT-ITFE feature distribution, EWT-TFE feature distribution and WTFE feature distribution, and for clarity, only four feature vectors of each mechanical condition are listed. Figure 15 shows that the characteristic curves of same type of signal have a certain degree of dispersion, however, the overall trend of these characteristic curves is similar. While the characteristic curves of different types of signals have obviously differences. Compared Figure 15a with Figure 15b,c, the superiorities of the proposed EWT-ITFE method are mainly reflected in the following aspects:

(1) It can be seen that the characteristic curves of same type of signal obtained by EWT-TFE and WTFE methods are more dispersed than the EWT-ITFE ones, this indicates that the EWT-ITFE method can better highlight the similarity between the same type of signal.
(2) It also can be seen that the overall trend of characteristic curves of different types of signals obtained by EWT-TFE and WTFE methods have a certain degree of similarity, especially the frequency domain features of Normal, Fault I and Fault II (as shown in Figure 15b,c). While for EWT-ITFE method, the characteristic curves between different types of signals are obvious different. These indicate that the EWT-ITFE method can better highlight the differences between different types of signals.

The differences between Figure 15a and Figure 15b are mainly caused by the division method of time-frequency plane. ITFE method divides the time-frequency plane according to the frequency distribution characteristics and energy distribution characteristics of the signal, while the TFE method has no basis for the division of the time frequency plane, besides, how to select the number of division boundary still lacks an effective criterion, usually we can only choose the number of division boundary by experience. Except for the division method of time-frequency plane, the differences between Figure 15a and Figure 15c are also caused by wavelet basis function and wavelet packet decomposition level. However, different from wavelet packet decomposition, EWT method can effectively extract the each vibration events of multi-component signals (as shown in Figure 5b), therefore, EWT-ITFE feature extraction method is adopted in this paper.

### 6.3. Classification by Using K-CV and GRNN

**Figure 16:**
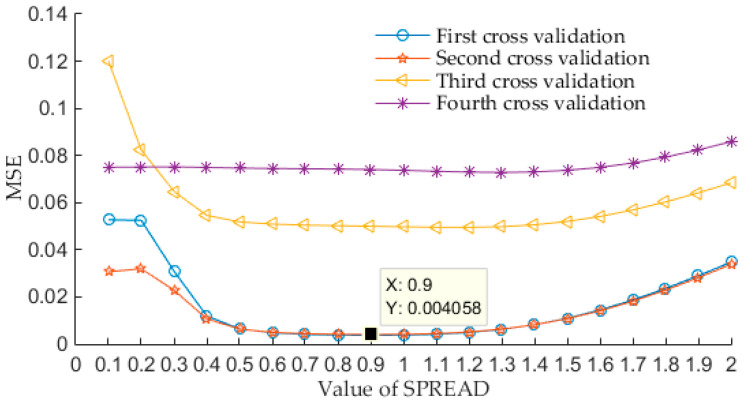
Four times optimization process of parameter “spread”.

According to the classifier design process shown in Figure 9, firstly, a total of 120 feature vectors should be randomly divided into test set and training set. For each type of signal, 30 feature vectors are include, we randomly select 20 feature vectors as the training samples, while other 10 feature vectors as test samples. Secondly, 2 fold cross validation is used in this paper, i.e., a total of 80 training samples are equally divided into 2 data sets again, one for training, while other for testing, and let cross validation performs four times. Besides, the loop traversal interval is [0.1, 2], and traversing step length *TL* is 0.1 in this paper, therefore, 20 “spread” values are available for selecting the optimal value. When the SME is minimum, the corresponding “spread” value is the best one. Figure 16 shows this parameter optimization process of 4 times cross-validation. For each cross validation process, we can see from Figure 16 that the value of “spread” has a tendency to decrease first and then increase, this indicates that the value of “spread” has a certain influence on the classification results, too large or too small “spread” values can’t make the performances of the classifier the best. Figure 16 shows that the minimum MSE value is obtained during the second cross validation process, at this time, the value of “spread” is 0.9.

A total of 40 feature vectors are entered into the optimal classifier (GRNN with “spread” = 0.9) for testing, while only 5 samples per fault type and 5 samples of normal condition are list in Table 3 as an example, the partial outputs of GRNN are shown in Table 3. Because there are four types of vibration signals, the number of output layer neuron in GRNN should be 4, i.e., the practical outputs of GRNN should be a series of four-dimensional vectors [y1, y2, y3, y4]. In Table 3, the expected vector [1, 0, 0, 0] represents Normal condition, expected vector [0, 1, 0, 0] represents Fault I condition, expected vector [0, 0, 1, 0] represents Fault II condition and expected vector [0, 0, 0, 1] represents Fault III condition, the closer the value between practical output vector and expected vector, the better the classification effect is. And usually, 0.5 is used as a threshold to judge the mechanical conditions, for example, if y1 is greater than 0.5, we think that the recognition result is Normal condition, and other cases are similar. Due to the large fluctuation of time-frequency entropy of some signals, it can be seen from Table 3 that only one fault I vibration signal is misidentified as a Normal signal, Finally, the vibration signal recognition rate are: Normal 100%, Fault I 90%, Fault II 100%, Fault III 100%.

In the past decades, RBFNN and BPNN are mostly widely used classifiers in HVCB fault diagnosis, and all these classifiers applied successfully in a certain extent, to prove the ability to distinguish normal and fault conditions of these classification methods, the GRNN, RBFNN and BPNN classifiers are compared in this paper, and the EWT-ITFE, EWT-TFE and WTFE feature extraction methods are adopted. GRNN classifier is optimized by the loop traversal method, and when using EWT-ITFE feature extraction method, the “spread” takes 0.9, while using other two feature extraction method, the “spread” takes 0.7, 1.2 respectively, as shown in Table 4.

The number of neurons in hidden layer have a certain effect on the prediction results of neural network. For RBFNN classifier, the number of neurons in input layer and output layer is 19, 4 respectively, while the number of neurons in hidden layer is determined by minimum prediction error of multiple trainings. For BPNN classifier, we followed the literature [3], BPNN is designed as a three-layer neural network in this paper, the number of neurons in input layer and output layer is the same as that in RBFNN, while the number of neurons in hidden layer are list in Table 4. Besides, the final recognition results of these three classifiers are shown in Table 4.

Table 4 shows that all samples of Fault III condition can be correctly identified by using each fault diagnosis method, while the samples of Fault I are easily misidentified, these indicate that the vibration signals of the fault III condition are very different from the vibration signals of the other three conditions, however, the vibration signals of the fault I condition is the opposite. Besides, for the same classifier, the EWT-ITFE feature vectors, EWT-TFE feature vectors and WTFE feature vectors are selected as the input vectors of the classifier respectively, Table 4 shows that the recognition rates of each type of signal are higher by using EWT-ITFE feature extraction method, this indicates that the signal features obtained by EWT-ITFE method are more significant. Compared optimal GRNN classifier with RBFNN and BPNN classifiers, Table 4 shows that using the same feature extraction method, the recognition rates of GRNN classifier are higher than other two classifiers. In general, Table 4 shows that the EWT-ITFE feature extraction method and the optimal GRNN classifier are more suitable for mechanical fault diagnosis of HVCBs. Only one parameter in GRNN needs to be optimized, and this parameter is easy to determine by using loop traversal method.

## 7. Conclusions

This paper presents a new method for mechanical fault diagnosis of HVCBs. Firstly, the original vibration signals are collected by designed acquisition system, and secondly, EWT is employed to decompose the vibration signals into a series of physically meaningful modes, and then, an improved TFE method is used for feature extraction. Finally, GRNN is employed to identify four mechanical conditions, where the parameter “spread” of GRNN is optimized by loop traversal method, meanwhile, K-CV is used to verify the performances of the optimal parameter spread. The simulation and practical test results demonstrate the following advantages of the new method:

(1) Compared with EMD, EEMD, IF and VMD methods, the EWT method can better decompose the signals into a series of physically meaningful modes, while the VMD method is difficult to determine the value of parameter *K* and other remain mentioned methods contain the false modes.
(2) Compared with EWT-TFE and WTFE feature extraction method, the proposed EWT-ITFE method is more superior in feature extraction. The feature curves of different types of signals obtained by EWT-ITFE method are more dispersed than other two method, this is good for classification.
(3) GRNN has the parameter spread needs to be determined, it usually depends on human choice. Combined with K-CV, the loop traversal method can effectively find the best value of parameter.
(4) According to the final recognition rates, the EWT-ITFE feature extraction method and the optimal GRNN classifier are more suitable for mechanical fault diagnosis of HVCBs, the signal features obtained by EWT-ITFE method are more significant, and the optimal GRNN classifier is more robust. The proposed EWT-ITFE method provides a new idea for mechanical feature extraction of HVCBs.
(5) According to the final recognition rates, the fault diagnosis results mainly depend on the feature extraction method and classifier.

## Figures and Tables

**Table 1:** Parameters of the simulation vibration signal.

Vibration Event $m_{i}$	$t_{i}$/ms	$f_{i}$/Hz	$A_{i}$	$\alpha_{i}$
$m_{1}$	15	1200	0.1	85
$m_{2}$	21	4000	0.3	95
$m_{3}$	25	5500	1.0	75
$m_{4}$	34	7000	0.5	60
$m_{5}$	51	2300	0.2	45

**Table 2:** Division boundaries of standard time-frequency plane.

Item	Division Boundaries										
frequency (Hz)	0	1475	2425	2675	2875	3175	3725	5825	12430	$f_{max}$	–
Serial number	1	30	49	54	58	64	75	117	249	400	–
time (ms)	0	23.23	24.05	24.75	26.4	33	35.15	37	39.78	46.11	200
sampling point	1	929	962	990	1056	1320	1406	1480	1591	1844	7998

**Table 3:** Partial outputs of optimal GRNN.

Types	Outputs							
	y1		y2		y3		y4	
	Expected	Practical	Expected	Practical	Expected	Practical	Expected	Practical
Normal	1	**0.8977**	0	0.0936	0	0.0080	0	0.0006
Normal	1	**0.9173**	0	0.0765	0	0.0057	0	0.0005
Normal	1	**0.9352**	0	0.0621	0	0.0024	0	0.0002
Normal	1	**0.9109**	0	0.0816	0	0.0070	0	0.0005
Normal	1	**0.9227**	0	0.0627	0	0.0136	0	0.0010
Fault I	0	0.0000	1	**0.9386**	0	0.0613	0	0.0001
Fault I	0	0.0001	1	**0.9423**	0	0.0566	0	0.0009
Fault I	0	0.0000	1	**0.9232**	0	0.0767	0	0.0002
Fault I	0	0.2902	1	**0.6511**	0	0.0512	0	0.0074
Fault I	0	**0.7006**	1	0.2401	0	0.0533	0	0.0060
Fault II	0	0.0027	0	0.0001	1	**0.9384**	0	0.0588
Fault II	0	0.0038	0	0.0002	1	**0.9322**	0	0.0638
Fault II	0	0.0024	0	0.0002	1	**0.9369**	0	0.0605
Fault II	0	0.0016	0	0.0001	1	**0.9327**	0	0.0656
Fault II	0	0.0011	0	0.0001	1	**0.9396**	0	0.0593
Fault III	0	0.0787	0	0.0004	0	0.0773	1	**0.8436**
Fault III	0	0.0841	0	0.0003	0	0.0722	1	**0.8434**
Fault III	0	0.0888	0	0.0002	0	0.0583	1	**0.8527**
Fault III	0	0.0497	0	0.0002	0	0.0945	1	**0.8555**
Fault III	0	0.0552	0	0.0001	0	0.0634	1	**0.8811**

**Table 4:** Final recognition results.

Classifier	Feature Extraction Method (Parameter)	Recognition Rate				
		Normal	Fault I	Fault II	Fault III	All Test Samples
GRNN	EWT-ITFE (spread = 0.9)	100%	90%	100%	100%	97.5%
	EWT-TFE (spread = 0.7)	90%	80%	100%	100%	92.5%
	WTFE (spread = 1.2)	80%	80%	100%	100%	90%
RBFNN	EWT-ITFE (number of neurons in hidden layer = 12)	100%	90%	90%	100%	95%
	EWT-TFE (number of neurons in hidden layer = 23)	90%	80%	90%	100%	90%
	WTFE (number of neurons in hidden layer = 31)	70%	70%	90%	100%	82.5%
BPNN	EWT-ITFE (number of neurons in hidden layer = 20)	100%	90%	80%	100%	92.5%
	EWT-TFE (number of neurons in hidden layer = 16)	70%	70%	90%	100%	82.5%
	WTFE (number of neurons in hidden layer = 19)	60%	70%	70%	100%	75%
